# Supplementary material for: Network of hotspot interactions cluster tau amyloid folds
Source: Nat Commun. 2023 Feb 16;14:895. doi: 10.1038/s41467-023-36572-3 (PMC9935906; doi:10.1038/s41467-023-36572-3)
Supplement: Supplementary file 1 — Supplementary Information [file 41467_2023_36572_MOESM1_ESM.pdf]

Supplementary information

Network of hotspots interactions cluster tau amyloid folds

Vishruth Mullapudi[1,4], Jaime Vaquer-Alicea[1,4], Vaibhav Bommareddy[1], Anthony R. Vega[1], Bryan D. Ryder[1, 2], Charles L. White III[1], Marc. I. Diamond[1], Lukasz A. Joachimiak[1, 3, #]

Supplementary Figures and Legends

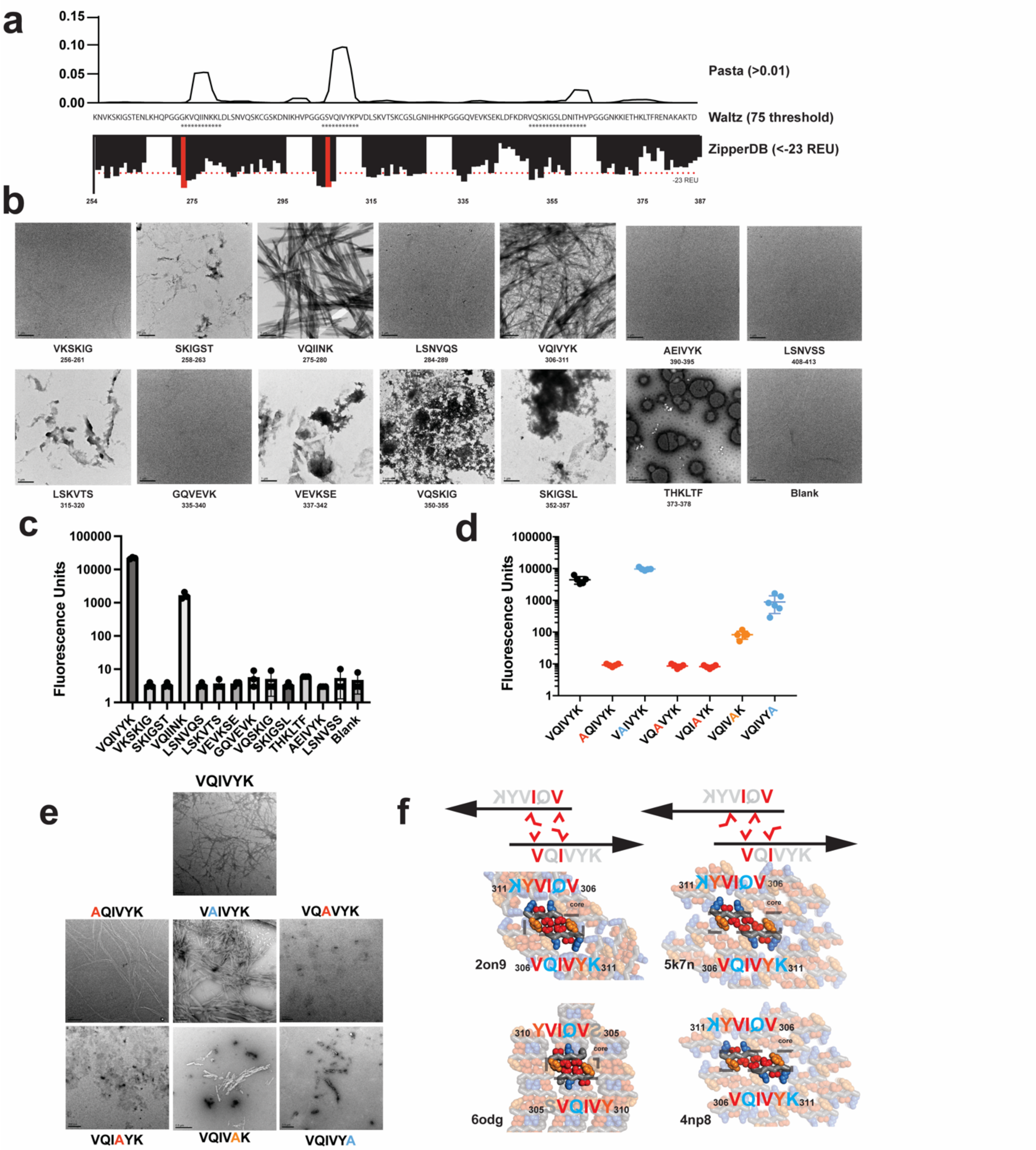

Supplementary Figure 1. Amyloid properties that determine assembly. (a) Prediction of amyloid motifs in tau using Pasta, Waltz and ZipperDB algorithms. Comparison across the methods uncovers VQIINK, VQIVYK and VQSKIG sequences as consensus motifs. Amyloid motifs in Pasta are shown as a profile (threshold >0.01) (top), Waltz predictions are shown by asterisk (middle) and ZipperDB hits are indicated as below -23 REU (dashed red line). **(b)** Representative TEM images of amyloid motifs from tau predicted by ZipperDB. Images were acquired from ThT fluorescence

aggregation endpoints from **(c)**. Scale bar is 1 μm. Imaging of the peptide aggregates using TEM was performed two independent times. **(c)** ThT fluorescence end points for predicted VQIVYK alanine mutants. Aggregation experiments were performed in triplicate and shown as averages with standard deviation. ThT peptide aggregation experiments were performed two independent times. **(d)** ThT fluorescence aggregation end points for ^{306}VQIVYK^{311} and alanine mutants at each position. Aggregation experiments were performed as five replicates and are shown as averages with standard deviation. Peptides that yielded high ThT are shown in blue, peptides with no ThT are colored in red and an intermediate mutant is colored in light blue. ThT peptide aggregation experiments were performed two independent times. **(e)** TEM images of VQIVYK and its alanine mutants. Scale bar is 1um. Imaging of the peptide aggregates using TEM was performed two independent times. **(f)** Mapping aggregation properties from the alanine mutants onto four available structures of VQIVYK (A-C; PDB IDs: 2n09, 5k7n and 4np4) or SVQIVY (D; PDB id 6odg). Symmetry related lattice of the peptide is shown in spacefill representation and the residues in the core “dimer” are stabilized key residues important for aggregation. The residues are colored by their aggregation signal from **(d)**. The structures are summarized by the relative register of the monomers in the core dimer, in register (left) or off set (right).

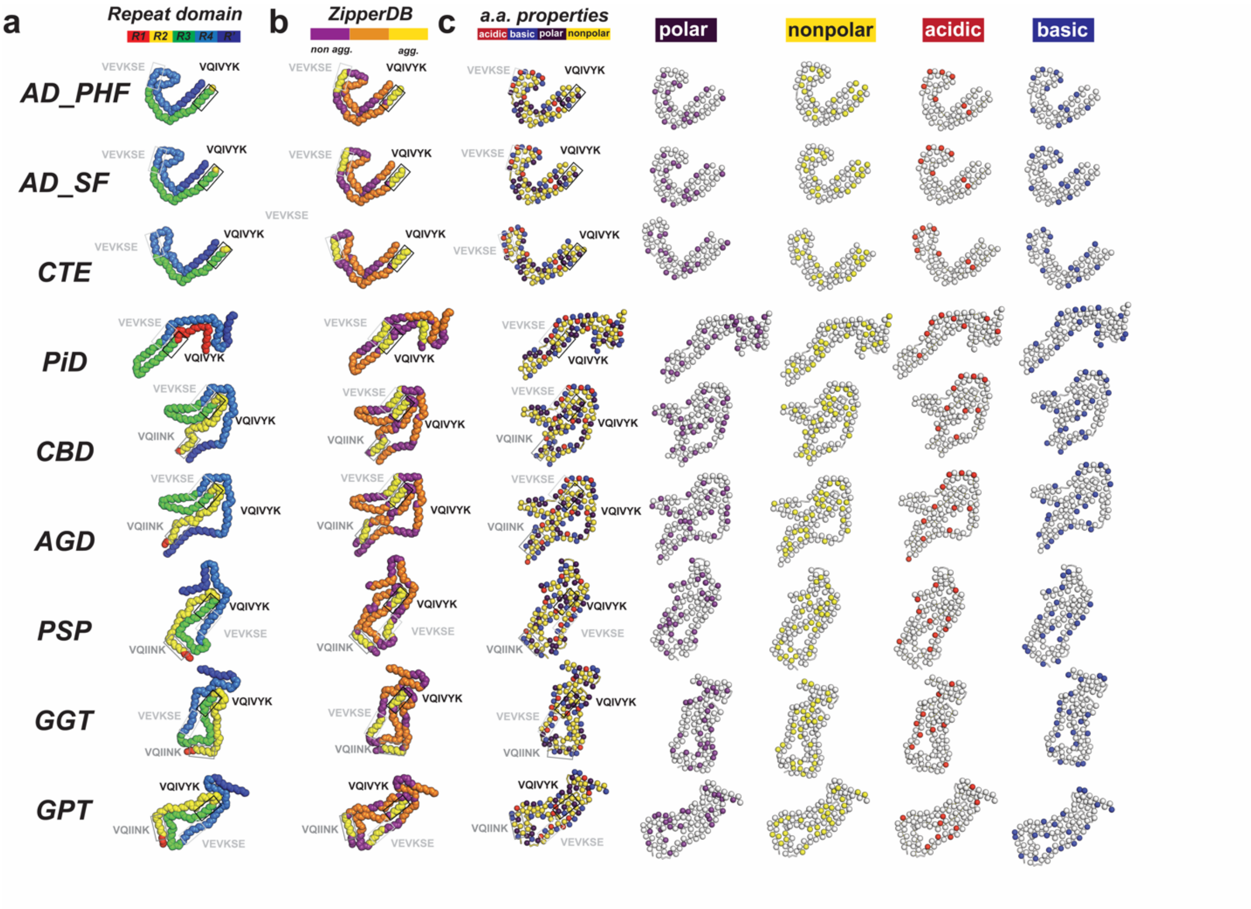

Supplementary Figure 2. Features of the tauopathy fibril structures. (a) Fibril structures are colored by repeat domain in red, yellow, green, blue and dark blue for repeat domains R1, R2, R3, R4, and R' respectively. Structures are shown in spacefill representation for the backbone. **(b)** Fibrils are colored by aggregation propensity of the different motifs colored in yellow, orange and magenta for high, medium, and low aggregation propensity. Structures are shown in spacefill for the backbone. **(c)** Fibrils are colored by amino acid type, nonpolar, polar, acidic, and basic are colored yellow, purple, red and blue respectively. Structures are shown using a ribbon representation and the c-β atom is shown in spheres. The ^{306}VQIVYK^{311}, ^{275}VQIINK^{280}, and ^{337}VEVKSE^{342} motifs are highlighted in a box in each structure. PDB ids: 5o3l, 5o3t 6gx5, 6nwp, 6tjo, 7p6d, 7p65, 7p66 and 7p6a were used for AD_PHF, AD_SF, CTE, PiD, CBD, AGD, PSP, GGT and GPT, respectively.

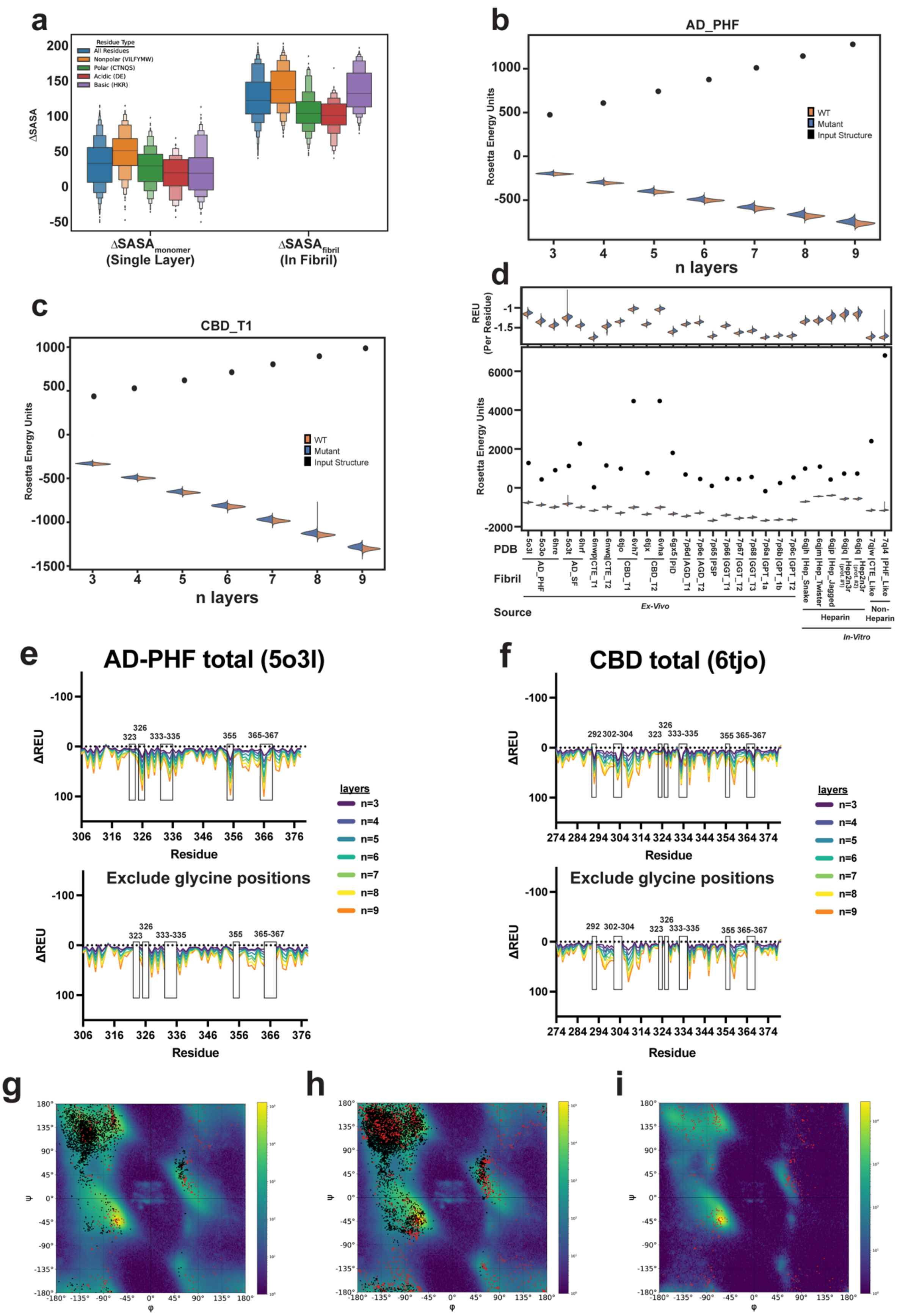
a
Residue Type
All Residues
Nonpolar (VILFYMW)
Polar (CTNQS)
Acidic (DE)
Basic (HKR)
ΔSASA
ΔSASA monomer (Single Layer)
ΔSASA fibril (In Fibril)
b
AD_PHF
Rosetta Energy Units
WT
Mutant
Input Structure
n layers
c
CBD_T1
Rosetta Energy Units
WT
Mutant
Input Structure
n layers
d
REU (Per Residue)
Rosetta Energy Units
PDB
Fibril
Source
5o3l AD_PHF
5o3o
6hre
5o3t AD_SF
6hrf
6nwp CTE_T1
6nwq CTE_T2
6tjo CBD_T1
6vh7
6tjx CBD_T2
6vha
6gx5 PiD
7p6d AGD_T1
7p6e AGD_T2
7p65 PSP
7p66 GGT_T1
7p67 GGT_T2
7p68 GGT_T3
7p6a GPT_1a
7p6b GPT_1b
7p6c GPT_T2
6qjh Hep_Snake
6qjm Hep_Twister
6qjp Hep_Jagged
6qjq Hep2n3r
7qjw CTE_Like
7ql4 PHF_Like
Ex-Vivo
Heparin
Non-Heparin
In-Vitro
e
AD-PHF total (5o3l)
ΔREU
Residue
Exclude glycine positions
323
326
333-335
355
365-367
f
CBD total (6tjo)
ΔREU
Residue
Exclude glycine positions
292
302-304
323
326
333-335
355
365-367
layers
n=3
n=4
n=5
n=6
n=7
n=8
n=9
g
h
i
ψ
φ

Supplementary Figure 3. Evaluating stability of fibril assemblies. (a) Solvent Accessible Surface Area (SASA) changes from the unfolded monomer to the folded monomer ($\Delta SASA^{\mathrm{monomer}}_{\mathrm{folding}}$) (left panel) or to the folded monomer in the fibril ($\Delta SASA^{\mathrm{in\ fibril}}_{\mathrm{folding}}$) (right panel) calculated for all, nonpolar, polar, basic, and acidic residues colored in blue, orange, green, red, and purple. Data is shown as a boxen or 'letter-value' plot for ∆SASA values for nine tauopathy protofilament structures. ΔSASA distributions are shown as letter-value plots with the center 2 boxes showing 50% of the data with each smaller box contain half of the remaining data. Total energies (REU) for AD-PHFs (PDB id: 5o3l) **(b)** and CBD (PDB id: 6tjo) **(c)** for WT and mutant assemblies across layers (n=3-9). Distribution of energies for WT and alanine mutants are shown in orange and blue, respectively. Energies for native structures are shown as black points. **(d)** Total energy distributions for minimized WT and mutant nine-mer (bottom), along with the energies of the input, un-minimized structures (points). Normalized distributions to the number of residues in the repeating monomer unit (top). The plot is colored as in **(b, c)**. PDB IDs: 7p6d, 7p6e, 6vha, 6vh7, 6tjo, 6tjx, 5o3l, 6hre, 7qjw, 6nwp, 6nwq, 6hrf, 7ql4, 5o3o, 5o3t, 7p65, 7p66, 7p67, 7p68, 6gx5, 7p6c, 7p6a and 7p6b. REU energy distributions are shown across 35 replicates at each position in each fibril and plotted as violin plots. **(e, f)** Energy perturbation at glycine positions when mutated to alanine. Energy profiles are shown for different layers (n=3-9) for AD-PHD and CBD and are colored from purple to orange. Glycine positions are numbered and indicated as boxes. **(g)** Overlay of φ/ψ torsional distributions for X-ray structures of globular proteins with resolution between 1.5 Å and 2.5 Å (110,677) to φ/ψ torsional angles for all residues (black) and glycine residues (red) in tau fibril structures (35 structures) or **(h)** all fibril structures (96) determined using helical reconstruction using RELION. **(i)** Overlay of alanine φ-ψ torsional distributions (background) for high resolution X-ray structures of globular proteins (1.5 - 2.5 Å) with glycine φ/ψ torsional angles (red) in tau fibrils.

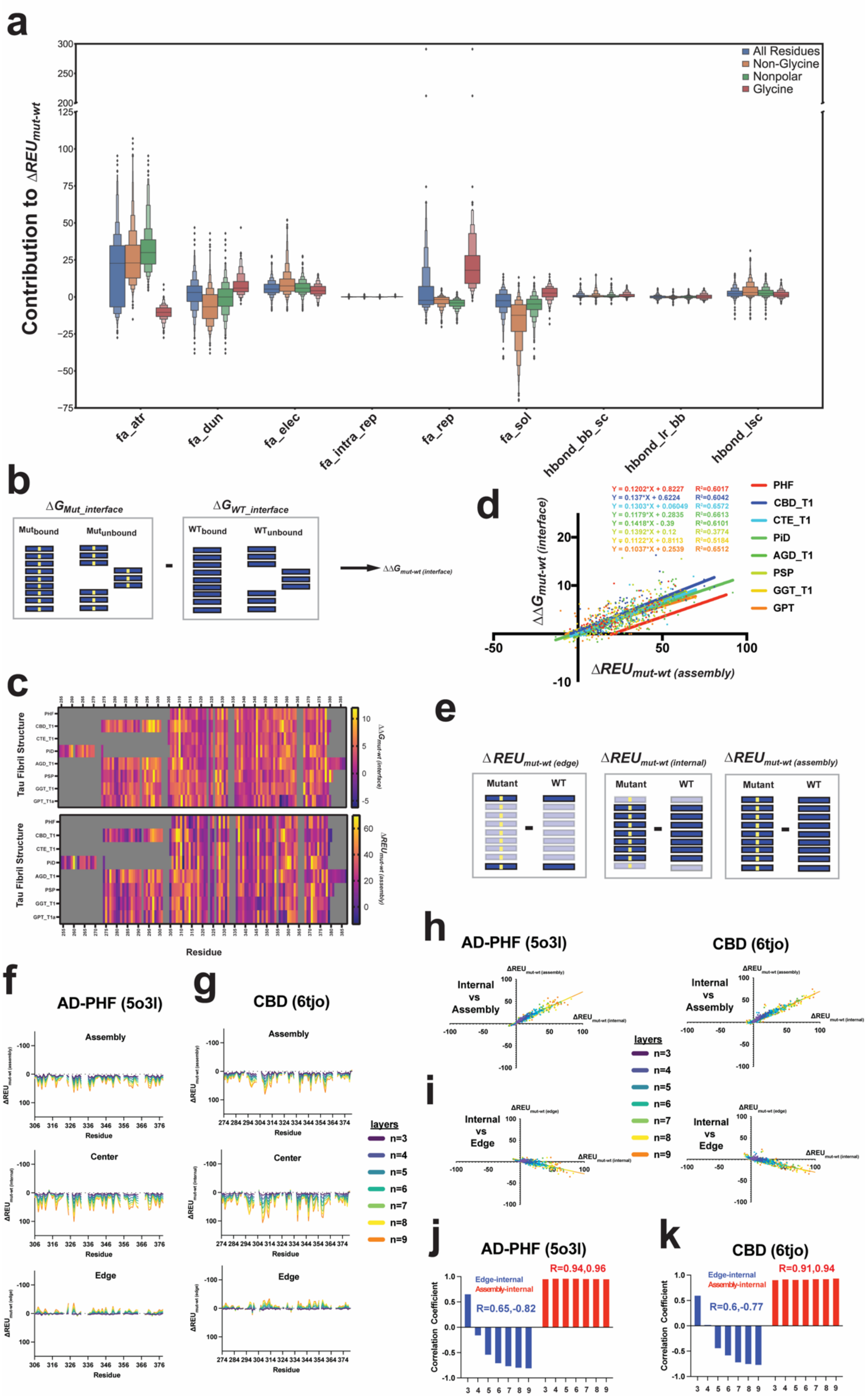
a
Contribution to ΔREU_mut-wt
All Residues
Non-Glycine
Nonpolar
Glycine
fa_atr
fa_dun
fa_elec
fa_intra_rep
fa_rep
fa_sol
hbond_bb_sc
hbond_lr_bb
hbond_lsc
b
ΔG_Mut_interface
ΔG_WT_interface
Mut_bound
Mut_unbound
WT_bound
WT_unbound
ΔΔG_mut-wt (interface)
c
Tau Fibril Structure
PHF
CBD_T1
CTE_T1
PiD
AGD_T1
PSP
GGT_T1
GPT_T1a
ΔΔG_mut-wt (interface)
ΔREU_mut-wt (assembly)
Residue
d
Y = 0.1202*X + 0.8227 R²=0.6017
Y = 0.137*X + 0.6224 R²=0.6042
Y = 0.1303*X + 0.06049 R²=0.6572
Y = 0.1179*X + 0.2835 R²=0.6613
Y = 0.1418*X - 0.39 R²=0.6101
Y = 0.1392*X + 0.12 R²=0.3774
Y = 0.1122*X + 0.8113 R²=0.5184
Y = 0.1037*X + 0.2539 R²=0.6512
PHF
CBD_T1
CTE_T1
PiD
AGD_T1
PSP
GGT_T1
GPT
ΔΔG_mut-wt (interface)
ΔREU_mut-wt (assembly)
e
ΔREU_mut-wt (edge)
ΔREU_mut-wt (internal)
ΔREU_mut-wt (assembly)
Mutant
WT
f
AD-PHF (5o3l)
g
CBD (6tjo)
Assembly
Center
Edge
Residue
layers
n=3
n=4
n=5
n=6
n=7
n=8
n=9
h
AD-PHF (5o3l)
CBD (6tjo)
Internal vs Assembly
i
Internal vs Edge
j
AD-PHF (5o3l)
k
CBD (6tjo)
Edge-internal
Assembly-internal
R=0.94,0.96
R=0.65,-0.82
R=0.91,0.94
R=0.6,-0.77
Correlation Coefficient

Supplementary Figure 4. Evaluation of per residue energetics in fibrils. (a) Breakdown of energy terms from Rosetta energy calculations evaluating their overall contribution to the assembly stability across nine different tauopathy fibril structures. Distributions for all, non-glycine, nonpolar and glycine are colored in blue, orange, green and red, respectively. Energy term distributions are shown across 35 replicates across positions clustered by amino acid properties. Energy distributions are shown as letter-value plots with the center 2 boxes showing 50% of the data with each smaller box contain half of the remaining data. Energy terms: fa_atr, fa_dun, fa_elec, fa_intra_rep, fa_rep, fa_sol, hbond_bb_sc, hbond_lr_bb, hbond_lsc correspond to full atom attractive, full atom Dunbrack rotamer, full atom electrostatic, full atom intramolecular repulsive, full atom repulsive, full atom solvation, hydrogen bonds backbone-side chain, hydrogen bonds backbone-backbone and hydrogen bonds side chain-side chain, respectively. **(b)** Schematic for the Flex ddG-based protocol for determining the contribution of residues to the energetics of inter-layer interfaces in protein fibrils. **(c)** Correlations between the *in silico* estimation of assembly stability upon mutation (x-axis) and the *in silico* "interface" inter-layer contribution of the assembly stability upon mutation (y-axis) for tauopathy fibrils. PDB ids: 5o3l, 6gx5, 6nwp, 6tjo, 7p6d, 7p65, 7p66 and 7p6a. **(d)** Heatmap comparison of the energetic change in response to substitution to alanine of the interface within a tau fibril (top) or of the total structural energy (bottom) as predicted by Rosetta. Scale colored in the plasma color scheme from yellow (most important) to purple (least important). **(e)** Schematic illustration for comparison of edge (left), internal (middle) and total (right) layer energetics in response to mutations. Comparison of total assembly (top), edge (middle) and internal (bottom) layer energetics as a function of layers for AD-PHF **(f)** and CBD **(g).** Profiles are shown for n=3 to n=9 layers and are colored from purple to orange. Correlation of energetics for total assembly to internal (top), edge to internal (middle) layers are shown for AD-PHF **(h)** and CBD **(i).** Correlation coefficients of edge to internal layer (blue) and total assembly to internal layer (red) energetics for AD-PHF **(j)** and CBD **(k).**

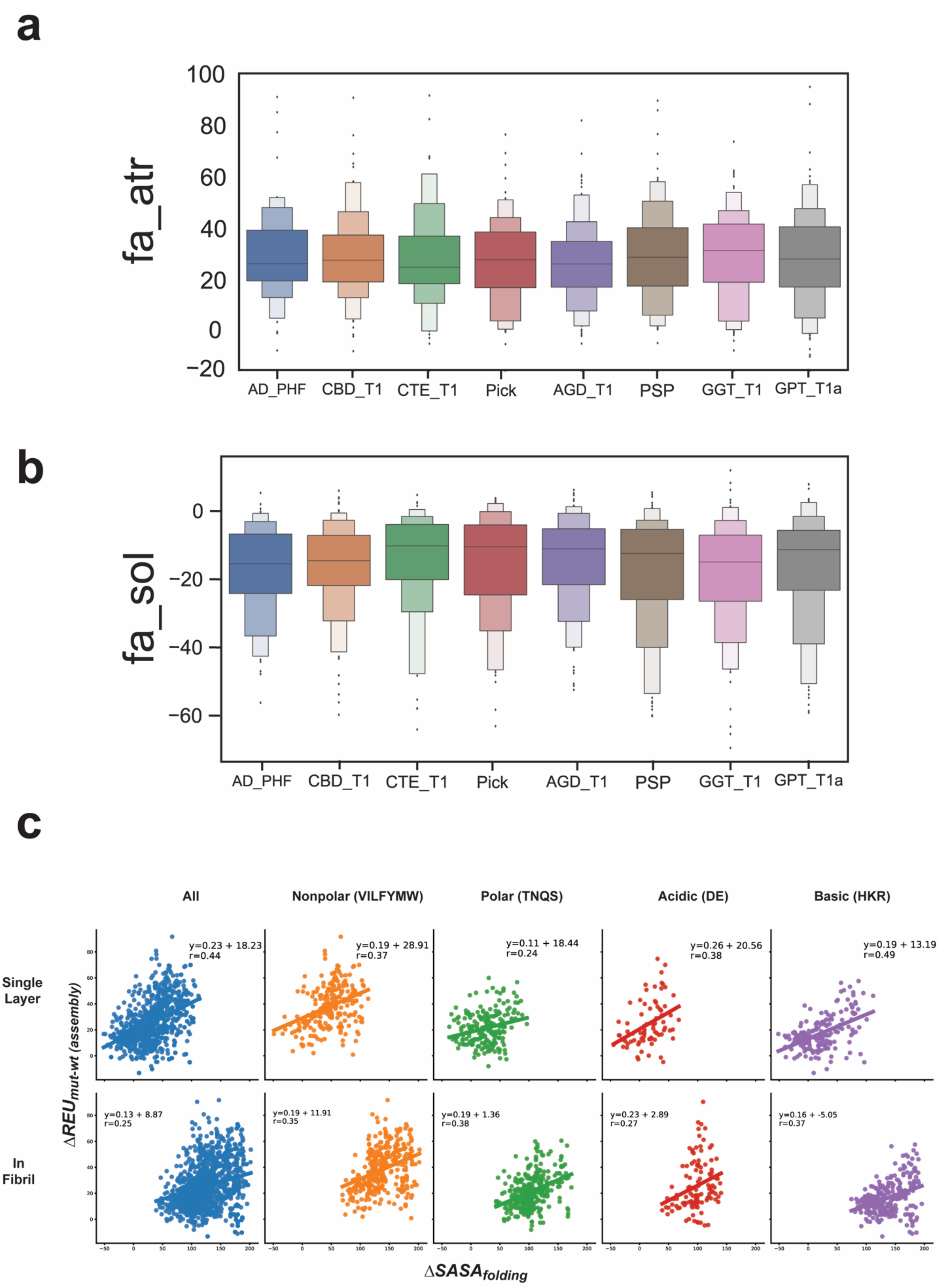
a
fa_atr
100
80
60
40
20
0
−20
AD_PHF
CBD_T1
CTE_T1
Pick
AGD_T1
PSP
GGT_T1
GPT_T1a
b
fa_sol
0
−20
−40
−60
AD_PHF
CBD_T1
CTE_T1
Pick
AGD_T1
PSP
GGT_T1
GPT_T1a
c
All
Nonpolar (VILFYMW)
Polar (TNQS)
Acidic (DE)
Basic (HKR)
Single Layer
In Fibril
ΔREU mut-wt (assembly)
y=0.23 + 18.23
r=0.44
y=0.19 + 28.91
r=0.37
y=0.11 + 18.44
r=0.24
y=0.26 + 20.56
r=0.38
y=0.19 + 13.19
r=0.49
y=0.13 + 8.87
r=0.25
y=0.19 + 11.91
r=0.35
y=0.19 + 1.36
r=0.38
y=0.23 + 2.89
r=0.27
y=0.16 + -5.05
r=0.37
ΔSASA folding

Supplementary Figure 5. Energy contributions and solvent accessibility to stabilizing fibril conformations. Comparison of the contribution of the full atom Lennard-Jones attractive potential energy (fa_atr) **(a)** and the full atom Lazaridis-Karplus solvation energy (fa_sol) (**b**) as calculated by Rosetta to the *in silico* predicted energy for the assembly of selected tauopathy fibril structures. Contributions are plotted in Rosetta Energy Units and are shown as letter-value plots with the center 2 boxes showing 50% of the data with each smaller box contain half of the remaining data. **(c)** Correlations between the ∆SASA of folding and the *in silico* change in assembly energy upon mutation (y-axis) for all, nonpolar, polar, acidic, and basic residues, either in the context of a single monomer folded into its fibril conformation without (top) or within (bottom) the context of the fibril. PDB ids: 5o3l, 6gx5, 6nwp, 6tjo, 7p6d, 7p65, 7p66 and 7p6a.

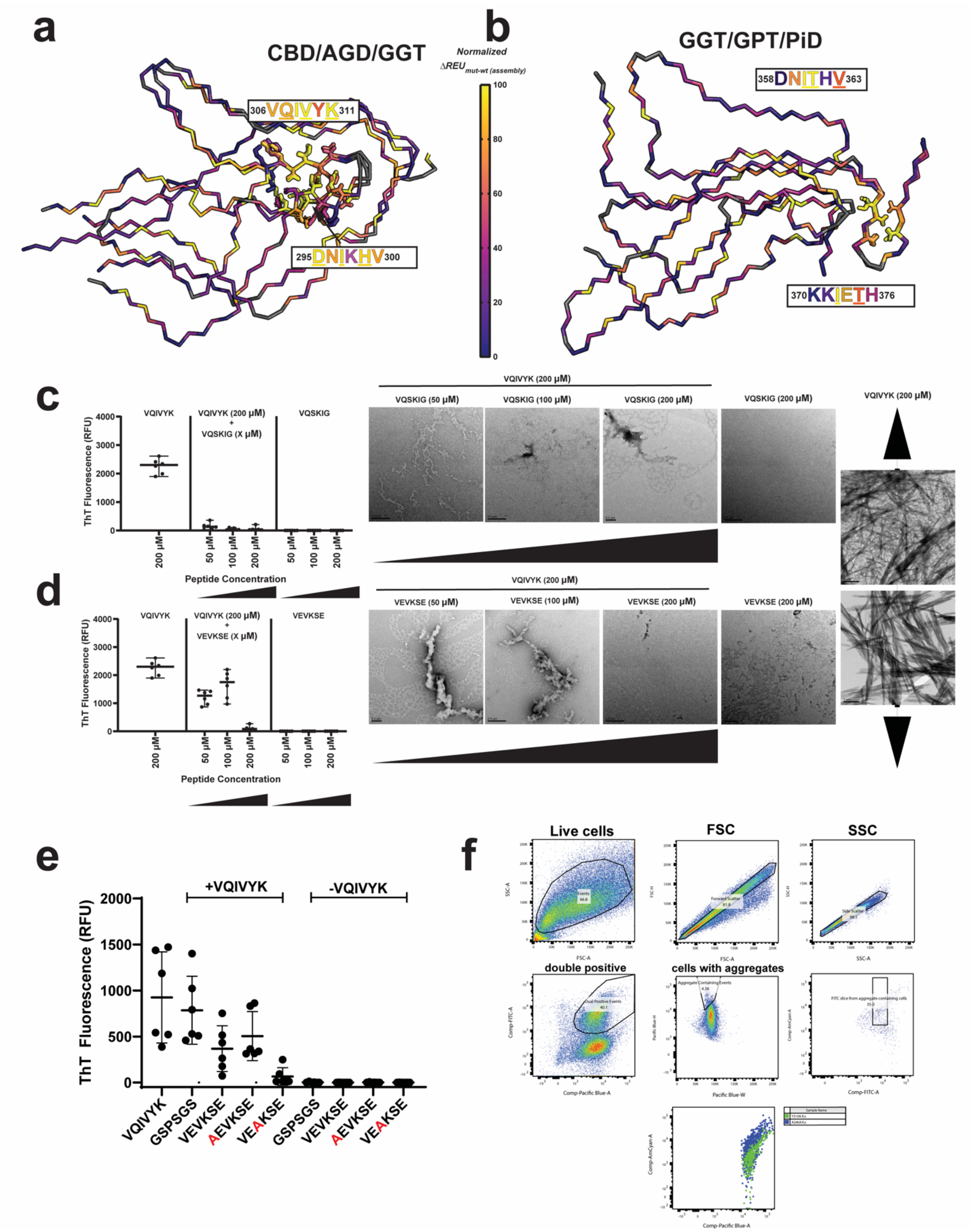

Supplementary Figure 6. Competing peptides interfere with VQIVYK assembly. (a) Alignment of CBD/AGD/GGT tauopathy fibril monomer conformations on the ^{306}VQIVYK^{311} amyloid-forming motif **(b)** and GGT/GPT/Pick's disease fibril monomer

conformations on ^{358}DNITHV^{363}. Residues colored in the plasma color scheme from yellow (important) to purple (unimportant) by their effect on *in silico* assembly stability when mutated to alanine. Structures shown as ribbons and key residues shown in stick representation. Endpoint Thioflavin T fluorescence values for 200 μM ^{306}VQIVYK^{311} peptide aggregation (left), 200 μM ^{306}VQIVYK^{311} with 50, 100 or 200 μM competitor (middle) and 50, 200 and 200 μM competitor alone (right). Competitor peptides ^{350}VQSKIG^{355} (**c**) and ^{337}VEVKSE^{342} (**d**) were co-aggregated with ^{306}VQIVYK^{311}. Experiments were performed as six technical replicates and are reported as averages with standard deviation. ThT peptide aggregation experiments were performed two independent times. TEM images of each endpoint sample shown on the right. Scale bars are shown as 0.2 μm. Imaging of the peptide aggregates using TEM was performed two independent times. (**e**) Aggregation of 50 μM ^{337}VEVKSE^{342}, VEVKSE alanine mutants (AEVKSE and VEAKSE) and GSPSGS control peptide with and without the presence of 200 μM ^{306}VQIVYK^{311} peptide. Experiment was performed as six technical replicates. Bars report average and standard deviation. ThT peptide aggregation experiments were performed two independent times. (**f**) Gating strategy for flow cytometry readout of in-cell incorporation assay on tau alanine mutants.

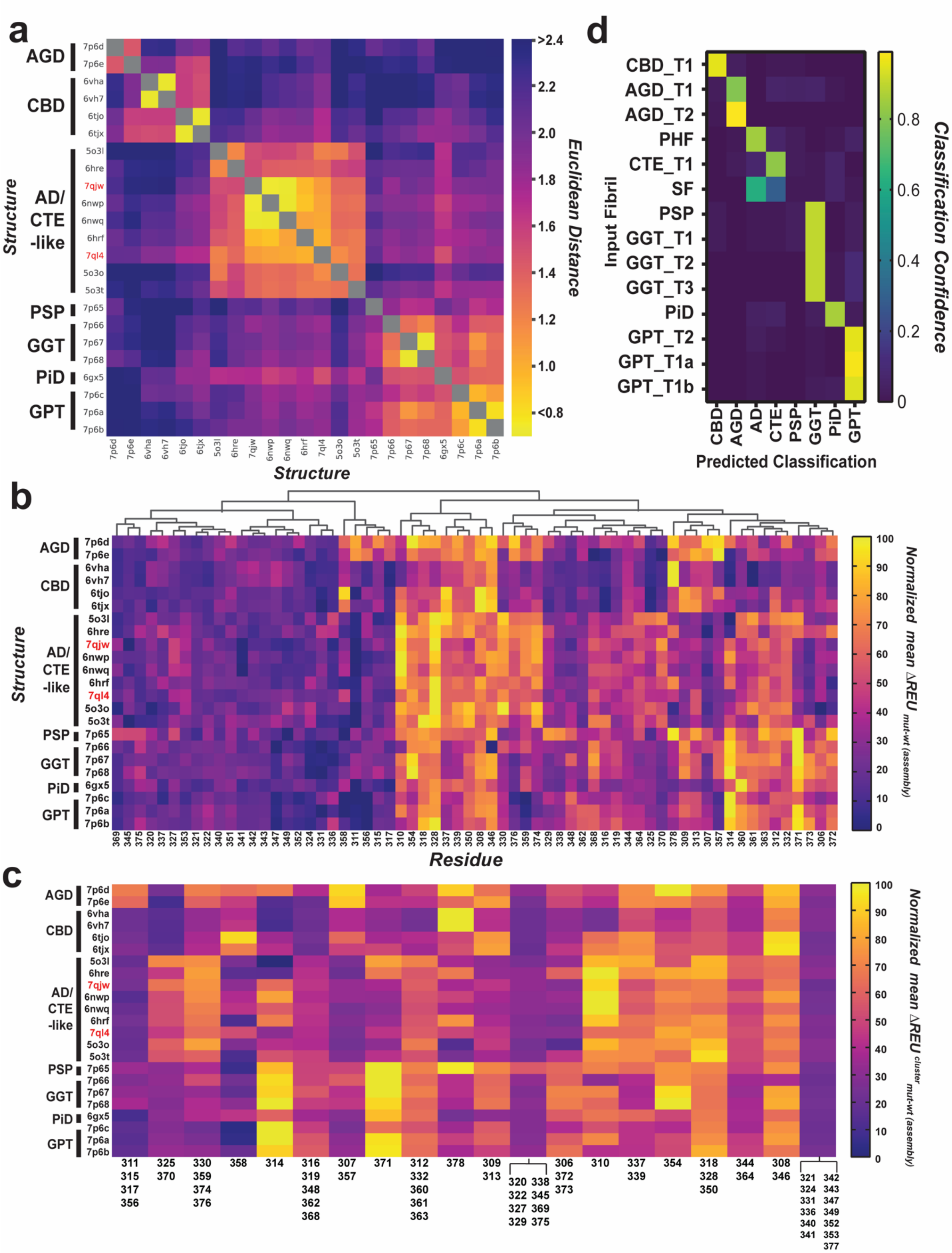

Supplementary Figure 7. ML-based classification of features based on residue energetics. (a) Similarity matrix of *ex vivo* fibrils based on *in silico*

$\Delta\mathrm{REU}_{\mathrm{mut-wt}}^{\mathrm{assembly}}$ measurements. Cells colored with the plasma color scheme by Euclidean distance between vectors of fibril pairs' $\Delta\mathrm{REU}_{\mathrm{mut-wt}}^{\mathrm{assembly}}$ measurements from yellow (<0.8 REU, most similar) to blue (>2.4, most dissimilar). **(b)** Dendrogram generated by hierarchical clustering via Ward's method of fibril residues, displayed alongside heatmap of *in silico* $\Delta\mathrm{REU}_{\mathrm{mut-wt}}^{\mathrm{assembly}}$. Dendrogram groups residues that covary by *in silico* $\Delta\mathrm{REU}_{\mathrm{wt-mut}}^{\mathrm{assembly}}$ across structures together. Residues are colored in the heatmap with the plasma color scheme from yellow (highest *in silico* $\Delta\mathrm{REU}_{\mathrm{mut-wt}}^{\mathrm{assembly}}$) to purple (lowest *in silico* $\Delta\mathrm{REU}_{\mathrm{mut-wt}}^{\mathrm{assembly}}$). **(c)** Heatmap of residue clusters generated via feature agglomeration, recursively combining the most similar residues/residue clusters together. Clusters are colored by the arithmetic mean value of residues comprising the cluster from yellow (highest mean *in silico* $\Delta\mathrm{REU}_{\mathrm{mut-wt}}^{\mathrm{assembly}}$) to purple (lowest mean *in silico* $\Delta\mathrm{REU}_{\mathrm{mut-wt}}^{\mathrm{assembly}}$). **(d)** Classification confidence matrix of the Random Forest Classifier when predicting classifications using the clustered mean *in silico* $\Delta\mathrm{REU}_{\mathrm{mut-wt}}^{\mathrm{assembly}}$ values derived from the *in silico* alanine scan. When given an input fibril structure's clustered mean *in silico* $\Delta\mathrm{REU}_{\mathrm{mut-wt}}^{\mathrm{assembly}}$ values, the squares represent the class probabilities the random forest classifier assigns to the input, colored with the plasma color scheme from yellow (1.0 probability, the classifier model is highly certain of a class assignment to the input) to purple (0 probability, the classifier model does not predict the input to belong to a given class). PDB IDs: 7p6d, 7p6e, 6vha, 6vh7, 6tjo, 6tjx, 5o3l, 6hre, 7qjw, 6nwp, 6nwq, 6hrf, 7ql4, 5o3o, 5o3t, 7p65, 7p66, 7p67, 7p68, 6gx5, 7p6c, 7p6a and 7p6b.
