# Supplementary material for: Network of hotspot interactions cluster tau amyloid folds
Source: Nat Commun. 2023 Feb 16;14:895. doi: 10.1038/s41467-023-36572-3 (PMC9935906; doi:10.1038/s41467-023-36572-3)
Supplement: Supplementary file 3 — Reporting Summary [file 41467_2023_36572_MOESM3_ESM.pdf]

nature portfolio

Corresponding author(s): Joachimiak, Lukasz A.

Last updated by author(s): Dec 20, 2022

Reporting Summary

Nature Portfolio wishes to improve the reproducibility of the work that we publish. This form provides structure for consistency and transparency in reporting. For further information on Nature Portfolio policies, see our Editorial Policies and the Editorial Policy Checklist.

Statistics

For all statistical analyses, confirm that the following items are present in the figure legend, table legend, main text, or Methods section.

n/a	Confirmed	
[ ]	[x]	The exact sample size (*n*) for each experimental group/condition, given as a discrete number and unit of measurement
[ ]	[x]	A statement on whether measurements were taken from distinct samples or whether the same sample was measured repeatedly
[x]	[ ]	The statistical test(s) used AND whether they are one- or two-sided<br>*Only common tests should be described solely by name; describe more complex techniques in the Methods section.*
[x]	[ ]	A description of all covariates tested
[x]	[ ]	A description of any assumptions or corrections, such as tests of normality and adjustment for multiple comparisons
[ ]	[x]	A full description of the statistical parameters including central tendency (e.g. means) or other basic estimates (e.g. regression coefficient) AND variation (e.g. standard deviation) or associated estimates of uncertainty (e.g. confidence intervals)
[ ]	[x]	For null hypothesis testing, the test statistic (e.g. *F*, *t*, *r*) with confidence intervals, effect sizes, degrees of freedom and *P* value noted<br>*Give P values as exact values whenever suitable.*
[x]	[ ]	For Bayesian analysis, information on the choice of priors and Markov chain Monte Carlo settings
[ ]	[ ]	For hierarchical and complex designs, identification of the appropriate level for tests and full reporting of outcomes
[x]	[ ]	Estimates of effect sizes (e.g. Cohen's *d*, Pearson's *r*), indicating how they were calculated

Our web collection on statistics for biologists contains articles on many of the points above.

Software and code

Policy information about availability of computer code

Data collection	Tau fibril dREU calculations were carried out with ROSETTA v3.12 (available at https://www.rosettacommons.org/). ThT data were acquired on a Tecan Spark platereader (https://lifesciences.tecan.com/multimode-plate-reader). FRET analysis of tau alanine mutants was acquired on a BD Fortessa flow cytometer. TEM images were acquired on a FEI Tecnai G2 Spirit Biotwin microscope.
Data analysis	All images of structures were produced in pymol v1.8.4.2. All plots were generated with python (v2.4) using Seaborn/Matplotlib or GraphPad Prism 9.4.1. FRET data was analyzed using FlowJo v10 (available at https://www.flowjo.com/solutions/flowjo/downloads).

For manuscripts utilizing custom algorithms or software that are central to the research but not yet described in published literature, software must be made available to editors and reviewers. We strongly encourage code deposition in a community repository (e.g. GitHub). See the Nature Portfolio guidelines for submitting code & software for further information.

Data

Policy information about availability of data

All manuscripts must include a data availability statement. This statement should provide the following information, where applicable:

- Accession codes, unique identifiers, or web links for publicly available datasets
- A description of any restrictions on data availability
- For clinical datasets or third party data, please ensure that the statement adheres to our policy

The ThT aggregation data and ΔREU_(mut-wt)^assembly data for tau fibrils generated in this study are available in the zenodo database under accession code 7275238 [https://zenodo.org/record/7275238]. PBD id's used in this study are: 7p6d [http://doi.org/10.2210/pdb7P6D/pdb], 7p6e [http://doi.org/10.2210/

pdb7P6E/pdb], 6vha [http://doi.org/10.2210/pdb6VHA/pdb], 6vh7 [http://doi.org/10.2210/pdb6VH7/pdb], 6tjo [http://doi.org/10.2210/pdb6TJO/pdb], 6tjx [http://doi.org/10.2210/pdb6TJX/pdb], 5o3l [http://doi.org/10.2210/pdb5O3L/pdb], 5o3t [http://doi.org/10.2210/pdb5O3T/pdb], 6hre [http://doi.org/10.2210/pdb6HRE/pdb], 7qjw [http://doi.org/10.2210/pdb7QJW/pdb], 6nwp [http://doi.org/10.2210/pdb6NWP/pdb], 6nwq [http://doi.org/10.2210/pdb6NWQ/pdb], 6hrf [http://doi.org/10.2210/pdb6HRF/pdb], 7ql4 [http://doi.org/10.2210/pdb7QL4/pdb], 5o3o [http://doi.org/10.2210/pdb5O3O/pdb], 7p65 [http://doi.org/10.2210/pdb7P65/pdb], 7p66 [http://doi.org/10.2210/pdb7P66/pdb], 7p67 [http://doi.org/10.2210/pdb7P67/pdb], 7p68 [http://doi.org/10.2210/pdb7P68/pdb], 6gx5 [http://doi.org/10.2210/pdb6GX5/pdb], 7p6c [http://doi.org/10.2210/pdb7P6C/pdb], 7p6a [http://doi.org/10.2210/pdb7P6A/pdb], 7p6b [http://doi.org/10.2210/pdb7P6B/pdb], 6qjh [http://doi.org/10.2210/pdb6QJH/pdb], 6qjm [http://doi.org/10.2210/pdb6QJM/pdb], 6qjp [http://doi.org/10.2210/pdb6QJP/pdb] and 6qjq [http://doi.org/10.2210/pdb6QJQ/pdb].

Human research participants

Policy information about studies involving human research participants and Sex and Gender in Research.

Reporting on sex and gender	N/A
Population characteristics	N/A
Recruitment	N/A
Ethics oversight	All of the research performed in this study complies with ethical regulations. All human samples utilized in these experiments were derived from deceased subjects. The UT Southwestern Institutional Review Board has determined that such studies are exempt from human subjects regulations as codified in federal law. All autopsies were performed only with permission of the decedents' next of kin or other person(s) legally authorized to provide such permission. All human materials utilized in these experiments were deidentified and no individually identifiable protected health information was available to the investigators using these materials.

Note that full information on the approval of the study protocol must also be provided in the manuscript.

Field-specific reporting

Please select the one below that is the best fit for your research. If you are not sure, read the appropriate sections before making your selection.

[x] Life sciences [ ] Behavioural & social sciences [ ] Ecological, evolutionary & environmental sciences

For a reference copy of the document with all sections, see nature.com/documents/nr-reporting-summary-flat.pdf

Life sciences study design

All studies must disclose on these points even when the disclosure is negative.

Sample size	We did not study populations either of animals or humans, thus population sample size is not applicable. In the case of Rosetta dREU calculations we ran 35 independent replicates for each mutant (and wild-type) minimization which we and others have shown converges on a solution (Barlow et al. 2018). Using the biohpc computer cluster at UTSW this represented 2 weeks of computer time.
Data exclusions	No data were excluded in the analyses
Replication	Figure and Supplementary Figure 1. The ThT aggregation assay with amyloidogenic peptides was performed as technical triplicates and the endpoint fluorescence values were plotted as averages with standard deviation. ThT aggregation data for VQIVYK and alanine variants was performed as six replicates and the endpoint fluorescence values were plotted as averages with standard deviation. TEM images were collected twice. Raw ThT data is reported in Zenodo deposition.<br><br>Figure 2. N/A. Flow chart describing each step in the in silico alanine scan procotol.<br><br>Supplementary Figure 2. N/A. Illustration of 9 different fibrillar structures from AD-PHF, CBD_T1, CTE_T1, PiD, AGD_T1, PSP, GGT_T1a and GPT_T1 (PDB ids: 5o3l, 5o3t, 6gx5, 6nwp, 6tjo, 7p6d, 7p65, 7p66 and 7p6a) colored by repeat domain, zipperdb aggregation propensity and amino acid properties.<br><br>Figure 3. All Rosetta simulations (#layers from n=3-9) were carried out using 35 replicates to ensure convergence of energies as previously determined (Barlow et al. 2018). RMSD and energy distributions are shown for all 35 replicates for each mutant and wild-type trajectory. Rmsd and energy distributions were calculated for all structures produced (35 replicates) cumulatively for all alanine variants and matching wild-type structures as a function of layer. The minimal set of 9 structures from AD-PHF, CBD_T1, CTE_T1, PiD, AGD_T1, PSP, GGT_T1a and GPT_T1 (PDB ids: 5o3l, 5o3t, 6gx5, 6nwp, 6tjo, 7p6d, 7p65, 7p66 and 7p6a).<br><br>Supplementary Figure 3. Change in SASA of different amino acids were calculated on a single structure comparing to the unfolded state to a single layer or a single layer in the context of a fibril. All Rosetta simulations for AD-PHF and CBD (#layers from n=3-9) were carried out using 35 replicates to ensure convergence of energies as previously determined (Barlow et al. 2018). RMSD and energy distributions are shown for all 35 replicates for each mutant and wild-type trajectory. The same dREU calculations were performed for 9 layers for all 30 tau fibril structures across 35 replicates cumulatively for WT and alanine variants as total energies but also per residue average energies (PDB IDs: 7p6d, 7p6e, 6vha, 6vh7, 6tjo, 6tjx, 5o3l, 5o3t, 6hre, 7qjw, 6nwp, 6nwq, 6hrf, 7ql4, 5o3o, 5o3t, 7p65, 7p66, 7p67, 7p68, 6gx5, 7p6c, 7p6a,

7p6b, 6qjh, 6qjm, 6qjp and 6qjq). For AD-PHF and CBD n=3 to n=9 the energetics of alanine mutations to proline were highlighted as contributing negatively due to sterics. Phi/psi torsional distributions extracted from the pdb (110,677 entries between 1.5 to 2.5 Angstroms) compared to tau fibril structures (35 entries), all Relion fibril structures (96 entries). Alanine residue phi/psi distributions (110,677 entries between 1.5 to 2.5 Angstroms) to glycine distributions in tau fibril structures.

Figure 4. Mean dREU values were normalized for each structure and compared as a window across 5 residues (2 to the left and right of the center). Per residue mean dREU values were mapped onto a single layer monomer conformation. Distributions of mean energy terms (across 35 replicates) as function of amino acid type mutated to alanine for 8 representative fibril structures from each disease. (PDB ids: 5o3l, 6gx5, 6nwp, 6tjo, 7p6d, 7p65, 7p66 and 7p6a).

Figure 5. FRET assays with tau alanine mutants were performed in triplicate and are shown as an average for top and bottom hits. The normalized dREU and the normalized in-cell FRET incoporation values are reported in Zenodo deposition.

Supplementary Figure 4. Breakdown of energy terms for all fibril analyses by amino acids types. Comparison of dREU for 9-mer vs interface ddG score for the minimal set of 9 structures from AD-PHF, CBD_T1, CTE_T1, PiD, AGD_T1, PSP, GGT_T1a and GPT_T1 (PDB ids: 5o3l, 5o3t, 6gx5, 6nwp, 6tjo, 7p6d, 7p65, 7p66 and 7p6a). Edge layer, internal layer and total assembly energetic comparison for AD-PHF and CBD for n=3 to n=9 layer assemblies. Comparison of 1) total assembly and internal layers and 2) edge layer and internal assembly for AD-PHF and CBD. Pearson correlation coefficients comparing 1) total assembly and internal layers and 2) edge layer and internal assembly for AD-PHF and CBD.

Figure 6 .The clustered dREU data was split for a training and test set. A random subset of 9/35 replicates was used for training and 29/35 was used for the test dataset. A random forest classifier with 100 features was used to train, classify and score and this was repeated 2,500 times and the best scoring classifier was used. The energetics for the fibrils were derived from AD, CBD, AGD, PSP, GPT, GGT and PiD, including subtypes. PDB id: 7p6d, 7p6e, 6vha, 6vh7, 6tjo, 6tjx, 5o3l, 5o3t, 6hre, 7qjw, 6nwp, 6nwq, 6hrf, 7ql4, 5o3o, 5o3t, 7p65, 7p66, 7p67, 7p68, 6gx5, 7p6c, 7p6a and 7p6b. The all-replicate dREU dataset used in training is available in the Zenodo deposition.

Supplementary Figure 5. Full atom Lennard-Jones attractive and Lazaridus-karplus Solvation energy as a function of mean energy from 35 replicates for the mutants calculated from ex vivo structures isolated from AD, CBD, AGD, PSP, GPT, GGT and PiD, including subtypes (PDB id: 7p6d, 7p6e, 6vha, 6vh7, 6tjo, 6tjx, 5o3l, 5o3t, 6hre, 7qjw, 6nwp, 6nwq, 6hrf, 7ql4, 5o3o, 5o3t, 7p65, 7p66, 7p67, 7p68, 6gx5, 7p6c, 7p6a and 7p6b). Relationship between dSASA (single layer and fibril) and mean dREU across different amino acid types for PDB ids: 5o3l, 6gx5, 6nwp, 6tjo, 7p6d, 7p65, 7p66 and 7p6a.

Figure 7. N/A. Model for discrimination of different tau fibril structures derived from the cumulative data in the manuscript.

Supplementary Figure 6. The ThT co-aggregation of VQIVYK with other peptides was performed as six technical replicates and the endpoint fluorescence values were plotted as averages with standard deviation. TEM images were collected twice. Raw ThT data is reported in Zenodo deposition.

Supplementary Figure 7. A similarity matrix was calculated using dREU values from dREU across 24 fibril structures (see below). Hierarchical clustering using Wards method for fibril residues for the same set of 24 fibril structures. Feature agglomeration combining residues/features for using data from 24 fibrils. Classification confidence matrix of the Random Forest Classifier when predicting classifications using the clustered mean dREU from the in silico alanine scan. The energetic data was calculated from the following PDB IDs: 7p6d, 7p6e, 6vha, 6vh7, 6tjo, 6tjx, 5o3l, 6hre, 7qjw, 6nwp, 6nwq, 6hrf, 7ql4, 5o3o, 5o3t, 7p65, 7p66, 7p67, 7p68, 6gx5, 7p6c, 7p6a, 7p6b.

Randomization	No data were randomized in the analyses
Blinding	No data were blinded in the analyses

Reporting for specific materials, systems and methods

We require information from authors about some types of materials, experimental systems and methods used in many studies. Here, indicate whether each material, system or method listed is relevant to your study. If you are not sure if a list item applies to your research, read the appropriate section before selecting a response.

Materials & experimental systems

n/a	Involved in the study
☒	☐ Antibodies
☐	☒ Eukaryotic cell lines
☒	☐ Palaeontology and archaeology
☒	☐ Animals and other organisms
☒	☐ Clinical data
☒	☐ Dual use research of concern

Methods

n/a	Involved in the study
☒	☐ ChIP-seq
☐	☒ Flow cytometry
☒	☐ MRI-based neuroimaging

Eukaryotic cell lines

Policy information about cell lines and Sex and Gender in Research

Cell line source(s)	293T/17 [HEK293T/17] (ATCC CRL-1268)
Authentication	Cell lines were not authenticated
Mycoplasma contamination	Cells were confirmed to be free of mycoplasma contamination
Commonly misidentified lines (See ICLAC register)	No misidentified cell lines were used in this study

Flow Cytometry

Plots

Confirm that:

- [x] The axis labels state the marker and fluorochrome used (e.g. CD4-FITC).
- [x] The axis scales are clearly visible. Include numbers along axes only for bottom left plot of group (a 'group' is an analysis of identical markers).
- [x] All plots are contour plots with outliers or pseudocolor plots.
- [x] A numerical value for number of cells or percentage (with statistics) is provided.

Methodology

Sample preparation	HEK293 cells stably expressing tauRD fused C-terminally to mEOS3.2 were seeded with lysates using lipofectamine prepared from CBD patient brain material. A portion of the mEOS was converted with UV, the cells fixed with PFA and analyzed by FACS.
Instrument	BD Fortessa
Software	FlowJo v10
Cell population abundance	20,000 cells were analyzed for each condition in triplicate
Gating strategy	Gates were selected in this order: live cells, FSC singlets, SSC singles, FITC/mCherry positive cells, and FRET positive population

- [x] Tick this box to confirm that a figure exemplifying the gating strategy is provided in the Supplementary Information.
